# Neuropathological characterization of the cavitating leukoencephalopathy caused by COA8 cytochrome *c* oxidase deficiency: a case report

**DOI:** 10.3389/fncel.2023.1216487

**Published:** 2023-08-04

**Authors:** Alexandra Chapleau, Renée-Myriam Boucher, Tomi Pastinen, Isabelle Thiffault, Peter V. Gould, Geneviève Bernard

**Affiliations:** ^1^Child Health and Human Development Program, Research Institute of the McGill University Health Centre, Montreal, QC, Canada; ^2^Department of Neurology and Neurosurgery, McGill University, Montreal, QC, Canada; ^3^Centre Hospitalier Universitaire de Québec-Université Laval, Québec City, QC, Canada; ^4^Genomic Medicine Center, Children’s Mercy Hospital, Kansas City, MO, United States; ^5^Kansas City School of Medicine, University of Missouri, Kansas City, MO, United States; ^6^Department of Pathology and Laboratory Medicine, Children’s Mercy Hospital, Kansas City, MO, United States; ^7^Service d’anatomopathologie Hôpital de l’Enfant-Jésus du CHU de Québec-Université Laval, Québec City, QC, Canada; ^8^Department of Pediatrics, McGill University, Montreal, QC, Canada; ^9^Department of Human Genetics, McGill University, Montreal, QC, Canada; ^10^Division of Medical Genetics, Department of Specialized Medicine, McGill University Health Centre, Montreal, QC, Canada

**Keywords:** neuropathology, COA8-related leukoencephalopathy, COX deficiency, APOPT1, cavitating leukoencephalopathy, mitochondrial disorders, case report

## Abstract

COA8-related leukoencephalopathy is a recently described rare cavitating leukoencephalopathy caused by biallelic variants in the *COA8* gene. Clinically, it presents heterogeneously and usually follows a bi-phasic clinical course with a period of acute onset and regression, followed by stabilization, and in some cases, even subtle improvement. We present a 4-year-old boy with a homozygous 2.5 kilobase pair deletion in the *COA8* gene following a severe neurological deterioration resulting in death weeks after onset. Brain MRI revealed a distinctive pattern of cavitating leukodystrophy predominantly involving the posterior cerebral white matter which improved upon a follow-up MRI a month later. Brain pathology displayed overall white matter destruction with gliosis and infiltration by macrophages. There was preservation of astrocytes around blood vessels and axons around the zones of demyelination. This study is the first neuropathological examination of COA8-related leukoencephalopathy and provides further characterization of the clinical and MRI phenotype.

## 1. Introduction

Mitochondrial disorders with cytochrome *c* oxidase (COX) deficiency are a group of clinically variable disorders defined by dysfunction of complex IV, the 14-subunit terminal enzyme of the electron transport chain (ETC) responsible for catalyzing an oxidation-reduction reaction involving cytochrome *c* and oxygen (Tsukihara et al., 1996; Zong et al., 2018; Brischigliaro and Zeviani, 2021). They are caused by mutations in over 30 nuclear and mitochondrial genes encoding subunits of the enzyme and proteins involved in its assembly and regulation (Rak et al., 2016). Included is cytochrome *c* assembly factor 8 (COA8), previously referred to as APOPT1 (MIM:616003), an enzyme involved in biogenesis of complex IV as well as its protection from oxidative stress (Brischigliaro et al., 2019; Signes et al., 2019). Biallelic pathogenic variants in *COA8* cause a rare cavitating leukoencephalopathy (MIM:619061), with only 8 patients reported to date (Melchionda et al., 2014; Sharma et al., 2018; Hedberg-Oldfors et al., 2020). Clinically, COA8-related leukoencephalopathy has a heterogenous presentation ranging from a sudden loss of motor and cognitive skills in early childhood to mild neurological deficits in adolescence. Most patients develop developmental delay, spastic quadriparesis, seizures and sensorimotor polyneuropathy. Disease progression typically follows a bi-phasic course with a period of acute regression lasting 2 months to 2 years, followed by long-term stabilization, and in some cases, even subtle improvement (Melchionda et al., 2014; Hedberg-Oldfors et al., 2020). Despite the wide phenotypic variation, all patients present with a hallmark brain magnetic resonance imaging (MRI) pattern of a posterior predominant cavitating leukoencephalopathy (Melchionda et al., 2014; Sharma et al., 2018; Hedberg-Oldfors et al., 2020). Treatment for COA8-related leukoencephalopathy is supportive, although some patients have been reported to take a variety of vitamins and cofactors, the efficacy of which is unknown (Melchionda et al., 2014; Sharma et al., 2018; Hedberg-Oldfors et al., 2020). Here, we report for the first time the neuropathological findings associated with COX deficiency caused by a homozygous 2.5 kilobase pair deletion in *COA8*.

## 2. Case presentation

**FIGURE 1:**
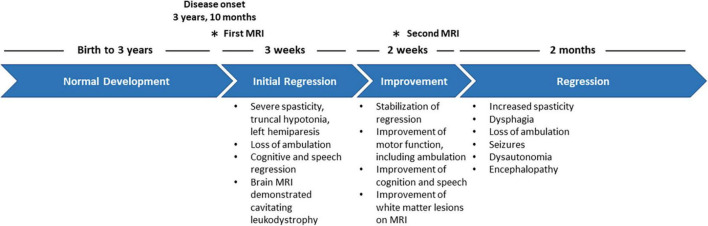
Clinical timeline depicting the disease progression of the patient from disease onset to mortality.

**FIGURE 2:**
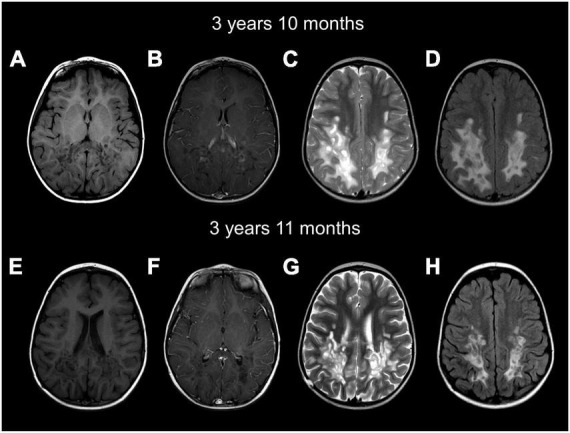
Brain MRIs at age 3 years and 10 months **(A–D)** and 3 years and 11 months **(E–H)** show a cavitating leukodystrophy with white matter abnormalities predominantly involving the posterior cerebral white matter and the splenium of the corpus callosum. Axial images show hypointense T1 **(A,B,E,F)** and hyperintense T2 **(C,G)** and FLAIR **(D,H)** parieto-occipital white matter. Numerous well-demarcated cysts are seen in the posterior white matter. Contrast imaging shows enhancement of the rim of cysts on the first MRI **(B)**. FLAIR images show the white matter involvement, together with the numerous well-demarcated cysts **(D,H)**. The follow-up MRI **(E–H)** shows mild improvement of the white matter abnormalities, less contrast enhancement and mild cerebral atrophy.

The patient, a male, was adopted and therefore no information was available regarding their prenatal, perinatal and family history. The patient had a normal development until age 3 years and 10 months, when they first presented with acute motor regression characterized by a loss of balance and decreased agility in both gross and fine motor skills, resulting in an increased number of falls while walking. Over a period of 3 weeks, the patient exhibited significant neurological and motor regression, developing progressive truncal hypotonia, severe spasticity and left hemiparesis, leading to the loss of ambulation. While cognition was normal at presentation, cognitive slowing was apparent during this period, and the patient also developed dysarthria, slowed speech and became more somnolent. This was followed by a 2-week period of improvement where the patient stabilized and recovered some lost function, including regaining ambulation and speech, and improved cognition. Then, rapid deterioration occurred, with increased spasticity and intense pain in the lower extremities, dysphagia, loss of ambulation, dystonic-like seizures, dysautonomia and encephalopathy, from which the patient succumbed within 2 months (Figure 1). The brain MRI at presentation (Figures 2A–D) revealed a distinctive pattern of a bilateral symmetric cavitating leukodystrophy, predominantly involving the posterior cerebral white matter and splenium of the corpus callosum. Axial T1-weighted, T2-weighted and FLAIR images displayed signal abnormalities of the affected white matter, including multiple well-demarcated cysts. After contrast injection, enhancement was seen, especially in the rim of the developing cystic lesions. Diffusion-weighted images showed multifocal areas of restricted diffusion. A follow-up MRI 2 weeks later (Figures 2E–H) showed improvement of the white matter lesions, as well as decreased contrast enhancement and diffusion restriction. Mild atrophy was noted. Recognition of an MRI pattern characteristic of a metabolic disease suggested a genetic etiology, prompting exome sequencing using genomic DNA extracted from patient blood (Thiffault et al., 2015). No candidate genes were detected; therefore, whole genome sequencing was pursued. A 2.5 kb deletion (chr14:103,573,453-103,575,949) was identified in the *COA8* gene (GRCh38/hg38), leading to the deletion of exon 3. This is predicted to cause a frameshift resulting in a truncated protein product (p.Glu121Valfs*4) (Supplementary Figure 1).

**FIGURE 3:**
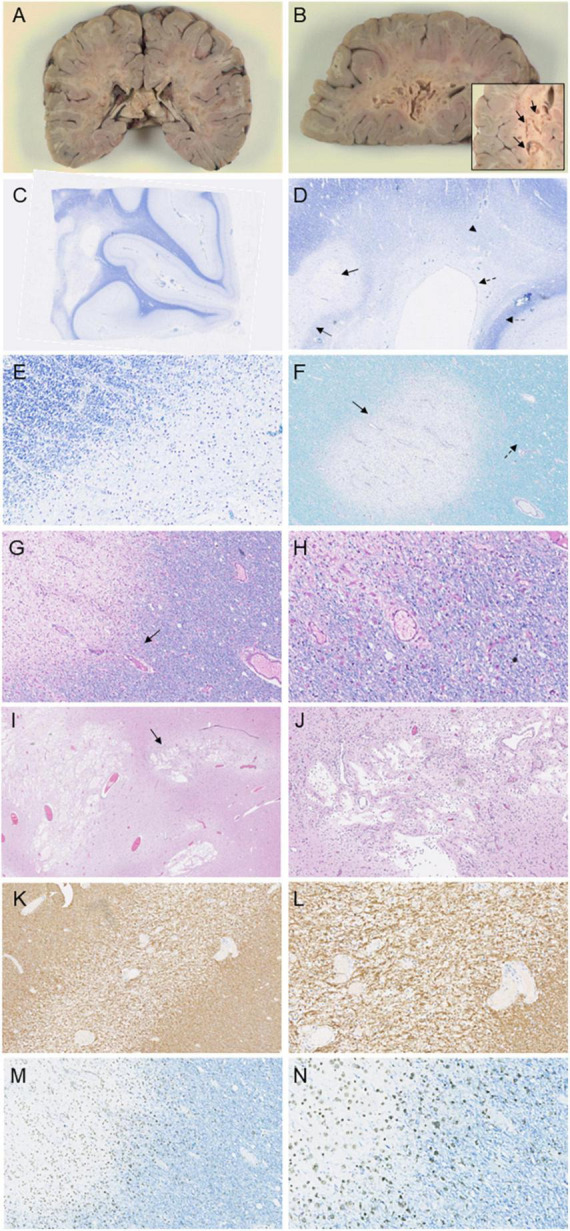
Immunohistochemical investigations of the patient following autopsy. Macroscopic images depict prominent lesions in coronal sections at the level of the splenium **(A)** and left occipital lobe **(B)**, including a magnified image of cavitations in the occipital white matter (arrow, **B**). Luxol staining of the occipital lobe **(C–E)** displays cavitation of the white matter (arrow, **D**) with surrounding well-circumscribed myelin loss (double-lined arrow, **D**). Diffuse myelin pallor is evident (arrowhead, **D**) as well as sparing of the ependyma (dashed arrow, **D**) and subcortical U-fibers (double-lined dashed arrow, **D**). Luxol PAS staining **(F)** and Luxol H&E staining **(G)** and magnified at 20× **(H)** in the frontal lobe reveal well-demarcated loss of myelin with rarefaction (arrow, **F**) and mild spongiosis (dashed arrow, **F**). Macrophages are evident within the rarefied region (arrow, **G**). H&E staining in the parietal lobe and splenium show cavitation in demyelinated areas (arrow, **I**), seen magnified at 10× **(J)**. Immunohistochemical staining of the temporal lobe and hippocampus with GFAP **(K)**, seen magnified at 20× **(L)** demonstrates reactive astrocytes near the demyelinated section. Staining of the frontal lobe with CD68 reveals macrophage invasion at the edge of the zones of more recent myelin loss **(M)**, and seen magnified at 20× **(N)**.

Neuropathological examination revealed prominent white matter abnormalities in the parietal and occipital lobes, including cystic lesions in the deep white matter (Figures 3A, B). Histological examination revealed widespread loss of myelin in the deep white matter of the cerebral hemispheres extending beyond the cystic changes noted on macroscopic examination (Figures 3C–J). Well demarcated lesions with cavitation were found in a serpiginous pattern throughout the parietal and occipital lobes, corresponding to the lesions noted on macroscopic examination (Figures 3C–E, I, J). Specifically, occipital sections showed clear cavitation of the white matter and diffuse myelin pallor with sparing of the ependyma (Figures 3C–E). The cerebral peduncles displayed focal myelin loss, consistent with a loss of occipito-bulbar projections. Decreased luxol staining was observed in the globus pallidus. White matter rarefaction was evident in the temporal lobe and hippocampus as well as in the frontal lobe, accompanied with mild spongiosis (Figures 3F–H). Immunostaining for CD68 revealed infiltration of macrophages at the edge of the zones of more recent demyelination (Figures 3M, N). GFAP staining of the temporal lobe showed preservation of astrocytes around blood vessels and reactive astrocytes at the edge of the demyelinated rarefied zone (Figures 3K, L). There was preservation of axons around the zones of demyelination although some mild spongiosis was seen around some well circumscribed demyelinated lesions. No neuronal perikarya was detected nearby areas of demyelination and there was no significant perivascular inflammation. Histological examination of the rest of the brain, cerebellum, brainstem and spinal cord was unremarkable.

## 3. Discussion

Integration of the clinical, radiological, and pathology data give insights into the pathophysiological mechanisms at play in COA8-related leukoencephalopathy. An acute disease onset seen in COA8 patients is a common finding in mitochondrial leukoencephalopathies, usually coinciding with a period of increased metabolic demand that cannot be met due to mitochondrial dysfunction (Baden et al., 2007; Melchionda et al., 2014; Rak et al., 2016; Murayama et al., 2019; Hedberg-Oldfors et al., 2020). Following the initial episode, many individuals recover, and brain development and maturation can continue, albeit with some damage, manifesting clinically as stabilization or improvement. Of the 8 previously reported patients, all had a sudden disease onset followed by a period of regression with 4 subjects demonstrating long-term stabilization of the disease and 3 showing subtle improvement. Only one other individual displayed a more severe and accelerated neurological deterioration culminating in death 6 months after onset, as seen in our patient (Sharma et al., 2018).

Two individuals have been previously reported with homozygous deletions of 12.82 kb and 12.41 kb, also resulting in loss of exon 3 (Melchionda et al., 2014; Sharma et al., 2018). This includes the only other severe patient as well as a more mildly affected individual with an improving phenotype (Melchionda et al., 2014; Sharma et al., 2018). Clinical variation is not atypical as patients with the same pathogenic variants can present with different phenotypes and disease evolution (Melchionda et al., 2014), suggesting that neurological deterioration in COA8 patients may be related to periods of environmental stress, a common finding in inherited metabolic disorders. Indeed, for 4 of 8 reported patients, disease onset coincided with a febrile illness (Melchionda et al., 2014; Sharma et al., 2018; Hedberg-Oldfors et al., 2020). Given the protective role of COA8 during redox imbalance, disease onset and further sequalae in these patients may therefore be related to periods of increased oxidative stress. A hypomorph COA8 may be unable to protect the COX complex from oxidative damage, thereby causing extreme biochemical impairment, and contributing to further oxidative stress. While there was no noticeable indication of major illness in our patient, it is possible that other stressors contributed to oxidative damage culminating in the severe clinical course.

The clinical, neuroimaging and neuropathological results presented in this study align with previous descriptions of patients with cavitating mitochondrial leukoencephalopathies caused by pathogenic variants encoding subunits of complex I, such as *NDUFV1* and *NDUFA1*. Neuropathological findings in both diseases revealed extensive myelin loss, numerous lesions with sparing of the basal ganglia, thalamus, cerebellum and brainstem, and macrophage infiltration at the sites of demyelination (Bindu et al., 2018; Becker et al., 2022). Notably, our patient demonstrated relative neuronal preservation near cavitated areas, consistent with these previous findings (Bindu et al., 2018; Becker et al., 2022). Furthermore, the preservation of subcortical U-fibers, a feature observed in our patient, has been documented in the pathological findings of a patient with *NDUFV1* variants and in individuals with a progressive cavitating leukoencephalopathy of unknown genetic etiology (Naidu et al., 2005; Becker et al., 2022). The absence of spinal cord abnormalities in our patient distinguishes them from those with *NDUFV1* variants, which involved the dorsal columns (Bindu et al., 2018; Becker et al., 2022).

Pathophysiological mechanisms may involve an initial disruption of the blood brain barrier, as indicated by enhancement on MRI and macrophage invasion, leading to the acute onset of clinical symptoms. Typical neurological signs associated with this disorder are consistent with the loss of white matter noted on the MRI and pathology analysis. Of interest is the relative neuronal preservation in areas of demyelination, suggesting that secondary axonal loss is not the primary driver of disease progression. Though the patient displayed a partially remitting MRI and clinical phenotype, the pathology demonstrated an overall loss of myelin and astrogliosis, indicating severe brain damage unable to support continued recovery and stabilization, especially in combination with the decreased capacity to relieve oxidative stress.

In conclusion, we report the second case with fulminant neurological deterioration resulting in early death, as well as the first neuropathological description of COA8-related leukoencephalopathy, shedding light on disease pathogenesis.

## Data availability statement

The datasets presented in this study can be found in online repositories. The names of the repository/repositories and accession number(s) can be found below: https://www.ncbi.nlm.nih.gov/clinvar/variation/2429110/?oq=SCV003798485&m=Single+allele, SCV003798485.

## Ethics statement

The studies involving human participants were reviewed and approved by the Research Ethic Boards of the Montreal Children’s Hospital and the McGill University Health Centre (11-105-PED, 2019-4972). Written informed consent to participate in this study was provided by the participants’ legal guardian/next of kin. Written informed consent was obtained from the individual(s), and minor(s)’ legal guardian/next of kin, for the publication of any potentially identifiable images or data included in this article.

## Author contributions

GB and AC conceived the study and wrote the original manuscript. IT and TP performed the genomic research analysis. PG performed the autopsy. PG and AC reviewed the autopsy findings. R-MB identified the patient. R-MB, GB, and AC completed the clinical phenotyping of the patient. All authors contributed to the editing and improving the manuscript.
