# Supplementary material for: Neuropathological characterization of the cavitating leukoencephalopathy caused by COA8 cytochrome c oxidase deficiency: a case report
Source: Front Cell Neurosci. 2023 Aug 4;17:1216487. doi: 10.3389/fncel.2023.1216487 (PMC10436302; doi:10.3389/fncel.2023.1216487)
Supplement: Supplementary file 1 [file Data_Sheet_1.docx]

Supplementary Material

Neuropathological characterization of the cavitating leukoencephalopathy caused by COA8 cytochrome *c* oxidase deficiency: a case report

**Alexandra Chapleau^1,2^, Renée-Myriam Boucher^3^, Tomi Pastinen^4,5^, Isabelle Thiffault^4,5,6^, Peter V. Gould^7^, Geneviève Bernard^1,2,8,9,10*^**

*** Correspondence:** Geneviève Bernard: genevieve.bernard@mcgill.ca

##
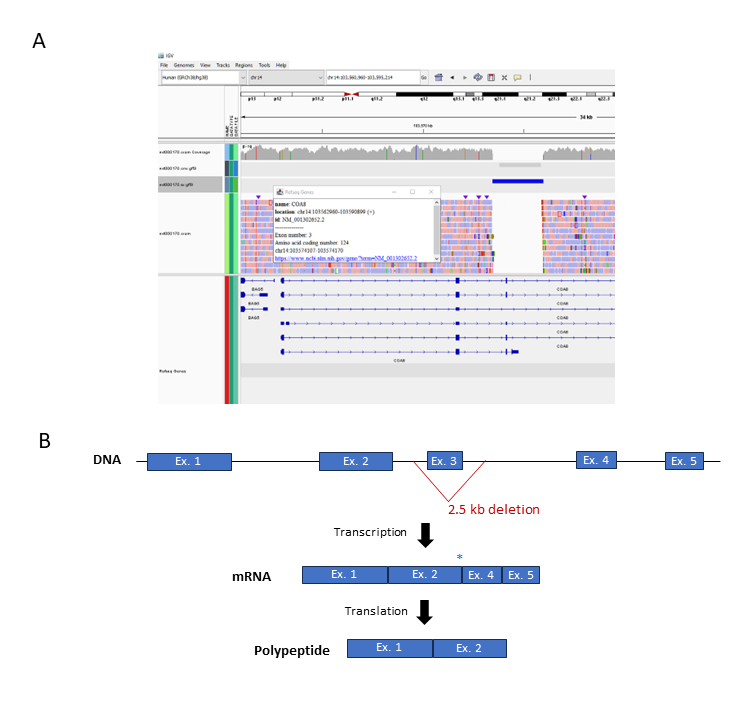
Supplementary Figures

**Supplementary Figure 1.** A homozygous 2.5 kb deletion in the COA8 gene was detected during whole genome sequencing, resulting in a loss of exon 3 (A). The loss of exon 3 causes a frameshift mutation resulting in an early stop codon, producing a truncated protein product of exons 1 and 2 (B).
